# Multi-omic analysis reveals the effects of interspecific hybridization on the synthesis of seed reserve polymers in a *Triticum turgidum* ssp. *durum* × *Aegilops sharonensis* amphidiploid

**DOI:** 10.1186/s12864-024-10352-9

**Published:** 2024-06-20

**Authors:** Qian Hu, Jing Liu, Xiaolei Chen, Carlos Guzmán, Qiang Xu, Yazhou Zhang, Qian Chen, Huaping Tang, Pengfei Qi, Mei Deng, Jian Ma, Guoyue Chen, Yuming Wei, Jirui Wang, Youliang Zheng, Yong Tu, Qiantao Jiang

**Affiliations:** 1 State Key Laboratory of Crop Gene Exploration and Utilization in Southwest China, Sichuan Agricultural University, Chengdu, Sichuan 611130 China; 2 Triticeae Research Institute, Sichuan Agricultural University, Chengdu, Sichuan 611130 China; 3 Departamento de Genética, Escuela Técnica Superior de Ingeniería Agronómica y de Montes, Universidad de Córdoba, Edificio Gregor Mendel, Campus de RabanaEles, Cordoba, 14071 Spain; 4 School of agricultural science, Xichang University, Xichang, Sichuan 615000 China

**Keywords:** *Aegilops Sharonensis*, *Triticum turgidum* ssp. *durum*, Amphidiploid, Multi-omics, Starch, Seed storage proteins

## Abstract

**Background:**

Wheat grain endosperm is mainly composed of proteins and starch. The contents and the overall composition of seed storage proteins (SSP) markedly affect the processing quality of wheat flour. Polyploidization results in duplicated chromosomes, and the genomes are often unstable and may result in a large number of gene losses and gene rearrangements. However, the instability of the genome itself, as well as the large number of duplicated genes generated during polyploidy, is an important driving force for genetic innovation. In this study, we compared the differences in starch and SSP, and analyzed the transcriptome and metabolome among *Aegilops sharonensis* (R7), durum wheat (Z636) and amphidiploid (Z636×R7) to reveal the effects of polyploidization on the synthesis of seed reserve polymers.

**Results:**

The total starch and amylose content of Z636×R7 was significantly higher than R7 and lower than Z636. The gliadin and glutenin contents of Z636×R7 were higher than those in Z636 and R7. Through transcriptome analysis, there were 21,037, 2197, 15,090 differentially expressed genes (DEGs) in the three comparison groups of R7 vs Z636, Z636 vs Z636×R7, and Z636×R7 vs R7, respectively, which were mainly enriched in carbon metabolism and amino acid biosynthesis pathways. Transcriptome data and qRT-PCR were combined to analyze the expression levels of genes related to storage polymers. It was found that the expression levels of some starch synthase genes, namely *AGP-L*, *AGP-S* and *GBSSI* in Z636×R7 were higher than in R7 and among the 17 DEGs related to storage proteins, the expression levels of 14 genes in R7 were lower than those in Z636 and Z636×R7. According to the classification analysis of all differential metabolites, most belonged to carboxylic acids and derivatives, and fatty acyls were enriched in the biosynthesis of unsaturated fatty acids, niacin and nicotinamide metabolism, one-carbon pool by folate, etc.

**Conclusion:**

After allopolyploidization, the expression of genes related to starch synthesis was down-regulated in Z636×R7, and the process of starch synthesis was inhibited, resulting in delayed starch accumulation and prolongation of the seed development process. Therefore, at the same development time point, the starch accumulation of Z636×R7 lagged behind that of Z636. In this study, the expression of the *GSe2* gene in Z636×R7 was higher than that of the two parents, which was beneficial to protein synthesis, and increased the protein content. These results eventually led to changes in the synthesis of seed reserve polymers. The current study provided a basis for a greater in-depth understanding of the mechanism of wheat allopolyploid formation and its stable preservation, and also promoted the effective exploitation of high-value alleles.

**Supplementary Information:**

The online version contains supplementary material available at 10.1186/s12864-024-10352-9.

## Background

Wheat grain is mainly composed of starch, protein and lipids, in which starch, as the main energy storage material, accounts for about 75% of the dry weight of the grain. Starch can be divided into amylose and amylopectin according to branching and its synthesis involves at least four enzyme families: adenosine diphosphate glucose pyrophosphorylase (AGPase), starch synthases (SSs), starch branching enzymes (SBEs), and starch debranching enzymes (DBEs) [1]. Studies have demonstrated that DBEs, such as isoamylase, play a role in starch synthesis and are essential for the formation of crystalline amylopectin [2]. Therefore, the expression of starch synthase genes determines the content and composition of starch [3].

The content of protein in wheat grain only accounts for about 15% of its dry weight, but it plays an important role in determining the processing quality of wheat flour. Seed storage proteins are mainly composed of high-molecular-weight glutenin subunits (HMW-GSs), low-molecular-weight glutenin subunits (LMW-GSs), and gliadins (α, β, γ and ω) [4]. HMW-GSs have been considered to be the most important seed storage proteins, and are well known to play a key role in determining wheat flour quality [5]. In wheat and its related wild species, the HMW-GS-encoding genes were the *Glu-1* loci, located on the long arms of group 1 chromosomes, with each locus comprising a pair of tightly linked genes encoding one x-type subunit with a higher molecular mass and one y-type subunit with a lower molecular mass [6]. As it evolved by artificial and natural selection, the diversity of HMW-GS in common wheat is relatively low, while there are abundant types of HMW-GS variation in wheat-related wild species, such as *Aegilops tauschii, Secale cereale, Aegilops umbellulata, Elymus glaucus, Elytrigia elongata* [7–11].

Polyploidization or whole genome duplication (WGD), a phenomenon in which the genome in the nucleus is doubled and passed on in a heritable manner to its offspring, is the driving force of plant evolution, and all seed plants have undergone this process throughout their evolutionary history [12, 13]. Polyploidy includes autopolyploidy and allopolyploidy. About 70% of flowering plants, including many important crops, are allopolyploids, such as common wheat [14], oilseed rape, *Brassica napus* [15], and cotton, *Gossypium hirsutum* [16]. Polyploidization results in duplicated chromosomes, and the genomes are often unstable and may result in a large number of gene losses and gene rearrangements [17]. Allopolyploids can act as important bridges by which to transfer valuable genes from wild crop ancestors into common wheat by hybridization of wheat and distantly related species to achieve wheat improvements. Therefore, studying the mechanism of allopolyploid formation and evolutionary variation is not only very important for basic research into plant evolution theory, but can also strengthen the directional selection of artificial allopolyploid evolution and has a huge value for wheat improvement.

*Aegilops sharonensis* (Eig) Á. Löve. (S^sh^S^sh^, 2n = 2x = 14) is an annual diploid grass species (Sharon goatgrass), which belongs to *Aegilops* section Sitopsis, is distributed within the Near East, and may have contributed to the B-genome of common wheat. It exhibits many excellent traits, including abiotic stress tolerance and biotic resistance to pests and diseases, such as stripe rust, stem rust, leaf rust, and powdery mildew [18, 19], representing a rich gene pool for wheat improvement. In our previous work, we characterized the novel HMW-GS variants 1S^sh^x2.9 and 1S^sh^y2.3 possessing much larger molecular weights than other known HMW-GSs in common wheat [20]. The *Glu-1S*^*sh*^ locus of *Ae. sharonensis* could be used for wheat quality improvement because the glutenin subunits encoded possess longer repetitive domains than most of the known subunits [21].

Prior to carrying out the current study, we successfully produced *T. turgidum* ssp. *durum* (durum wheat)-*Ae. sharonensis* amphidiploids by conducting wide hybridizations [22]. In the present study, we investigated the regulatory mechanism of changes in starch and storage protein contents in developing grains by transcriptome and metabolome analyses, to reveal the effects of interspecific hybridization and polyploidization on the synthesis of seed reserve polymers.

## Results

### Identification of HMW-GS composition and multi-color fluorescent in situ hybridization (mc-FISH) observation of the amphidiploid and its parents

**Fig. 1:**
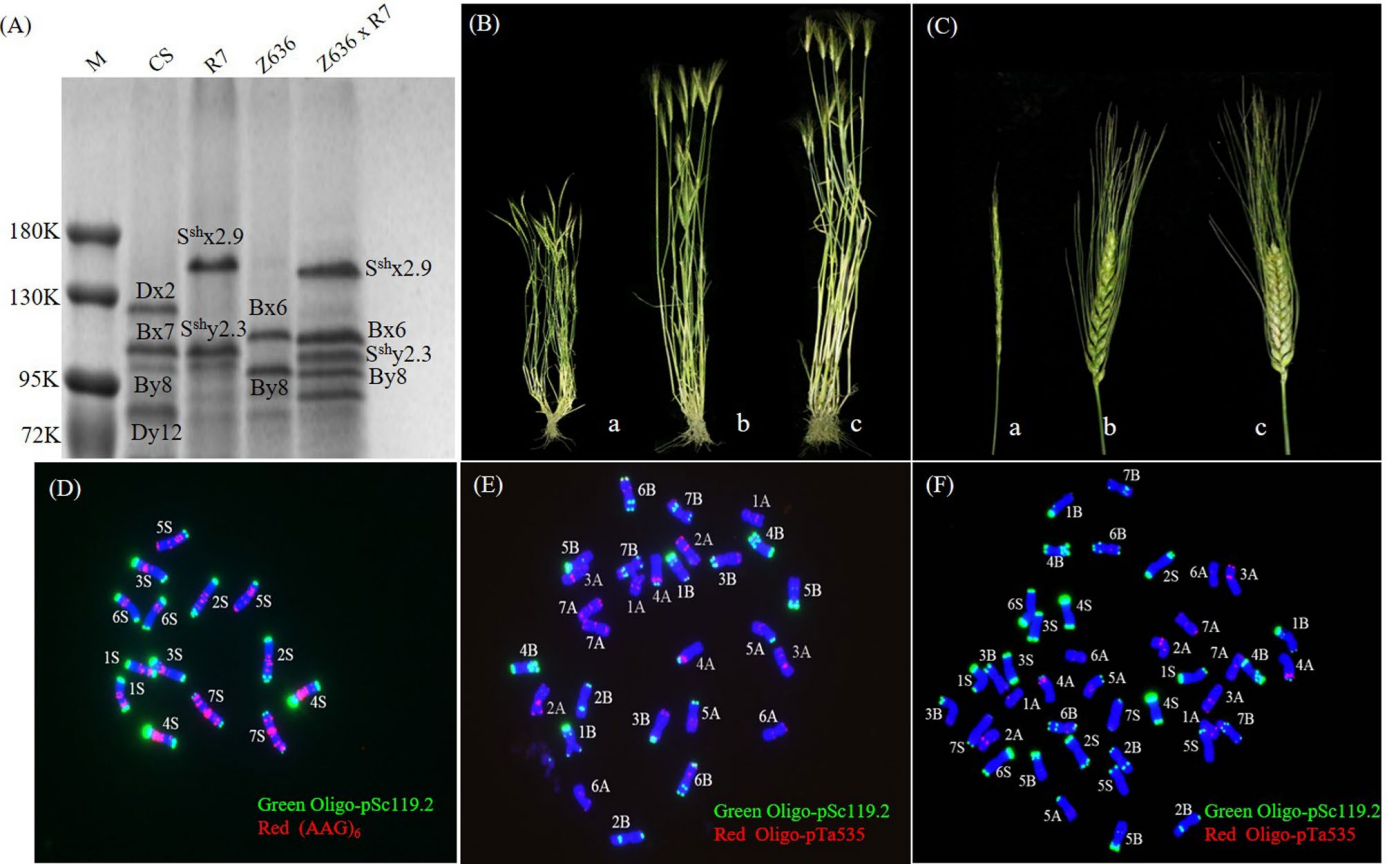
The morphology and cytology of R7, Z636, and Z636×R7. **A**, HMW-GS composition of R7, Z636 and Z636×R7 determined by SDS-PAGE (the full-length gel is included in Supplementary Fig. 1). **B**, **C**, Comparison of mature plant and spike morphology. Images a-c, R7, Z636, Z636×R7, respectively. **D**, The mc-FISH of R7 hybridized with probes Oligo-pSc119.2 (green) and (AAG_6_) (red). **E**, **F**. The mc-FISH of Z636 and Z636×R7 hybridized with probes Oligo-pSc119.2 (green) and Oligo-pTa535 (red)

The amphidiploid (Z636×R7, AABBS^sh^S^sh^, 2n = 42) was generated from a wide cross between *Triticum turgidum* ssp. *durum* (Z636, AABB, 2n = 28) and *Ae. sharonensis* (R7, S^sh^S^sh^, 2n = 14). The amphidiploid seeds with *Ae. sharonensis*-specific HMW-GS (1S^sh^x2.9 + 1S^sh^y2.3) and durum wheat-specific HMW-GSs (1Bx6 + 1By8) were identified by SDS-PAGE (Fig. 1A). The stem nodes and ears of the amphidiploid plants were light purple in color after the jointing stage, with the plant morphology showing a phenotype intermediate between the two parental types (Fig. 1B and C). Cytological observation of the three genotypes showed that the number of chromosomes in the amphidiploid was 42, which was composed of 14 *Ae. sharonensis* chromosomes (Fig. 1D) and 28 durum wheat chromosomes (Fig. 1E), so that the genetic composition was consistent with that expected, indicating that the amphidiploid has a complete set of durum wheat and *Ae. sharonensis* chromosomes (Fig. 1F).

### Content analysis of gluten proteins and starch

**Fig. 2:**
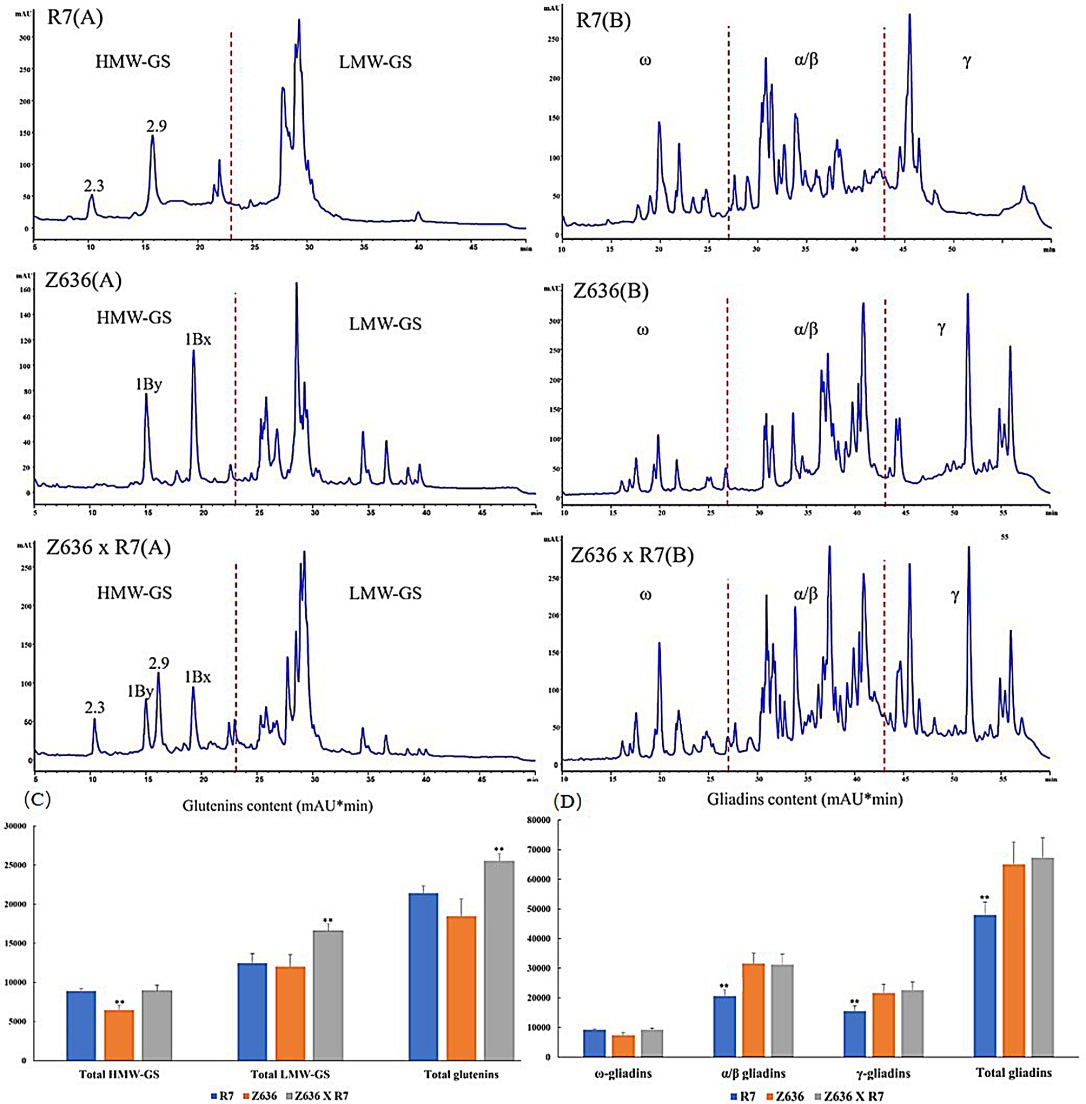
Contents of gluten protein and starch in the grains of R7, Z636, and Z636×R7. Asterisks indicate the statistical significance between R7, Z636, and Z636×R7 determined by one-way ANOVA (** at *p* < 0.01). **A**, Contents of glutenin subunits by RP-HPLC analysis. **B**, Contents of gliadins by RP-HPLC analysis. **C**, Comparison of relative contents of different glutenins. **D**, Comparison of relative contents of different gliadins

The contents of glutenin and gliadin of the three genotypes, namely R7, Z636, and Z636×R7, were analyzed by reverse-phase high-performance liquid chromatography (RP-HPLC) (Fig. 2A and B). R7 and Z636 each exhibited two peaks in the HMW-GS region, namely 1S^sh^x2.9 + 1S^sh^y2.3 and 1Bx6 + 1By8, respectively, whereas Z636×R7 exhibited all four peaks in the HMW-GS region (1Bx6 + 1By8, 1S^sh^x2.9 + 1S^sh^y2.3). The above results were consistent with the SDS-PAGE results, which confirmed that the HMW-GSs of R7 had been successfully introduced into the amphidiploid. The content of the HMW-GSs of Z636×R7 (8,928.87 mAU*min) was significantly higher than that in Z636 (6,472.57 mAU*min), but not significantly different from the content of HMW-GSs in R7 (8,935.36 mAU*min). The total LMW-GS content in Z636×R7 (16,590.25 mAU*min) was significantly higher than that in both R7 (12,482.67 mAU*min) and Z636 (12,009.62 mAU*min). Therefore, the total glutenin content in Z636×R7 (25,519.12 mAU*min) was significantly higher than that in R7 (21,418.03 mAU*min) and Z636 (18,482.19 mAU*min) (Fig. 2C).

Due to the small differences in molecular structure and molecular weight, the chromatographic peaks of α- and β-gliadins overlapped partially, so could not be separated from each other and could only be grouped as one class. There were differences in the peak shape and content of gliadins among the three genotypes; the contents of α-, β-, and γ-gliadins of R7 were significantly lower than those of Z636 and Z636×R7, and the total gliadin content of R7 (45,527.80 mAU*min) was significantly lower than the contents in Z636 (60,665.47 mAU*min) and Z636×R7 (63,186.04 mAU*min) (Fig. 2D).

The total starch and amylose contents of the three genotypes were determined in plants grown in the greenhouse (2019) and field (2021) (Table 1). In 2019, the contents of total starch and amylose of Z636×R7 (58.107%, 13.063%) were significantly lower than those of Z636 (61.124%, 35.545%), but higher than those of R7 (54.822%, 5.995%). Compared with 2019, the trend of change in amylose content in 2021 was consistent among the three materials, but the trend of total starch content was different, as the total starch content of Z636×R7 (61.163%) was higher than that of Z636 (58.272%), which may be due to environmental effects, resulting in the inconsistent trend of total starch content over the two years.

**Table 1:** Test of amylase and total starch contents of R7, Z636 and Z636×R7^a, b^

Lines	2019, Greenhouse		2021, Field	
	Total starch content (%)	Amylose content (%)	Total starch content (%)	Amylose content (%)
R7	54.822 ± 1.79c	5.995 ± 0.71c	42.455 ± 2.13c	25.262 ± 0.66b
Z636	61.124 ± 0.23a	35.545 ± 1.12a	58.272 ± 0.09b	30.114 ± 1.48a
Z636×R7	58.107 ± 1.81b	13.063 ± 1.64b	61.163 ± 1.32a	27.689 ± 2.31ab

^a^ Mean ± SD was calculated from triplicates

^b^ Values with different letters in the same column were significantly different with *P* < 0.05

### RNA-Seq analysis

**Fig. 3:**
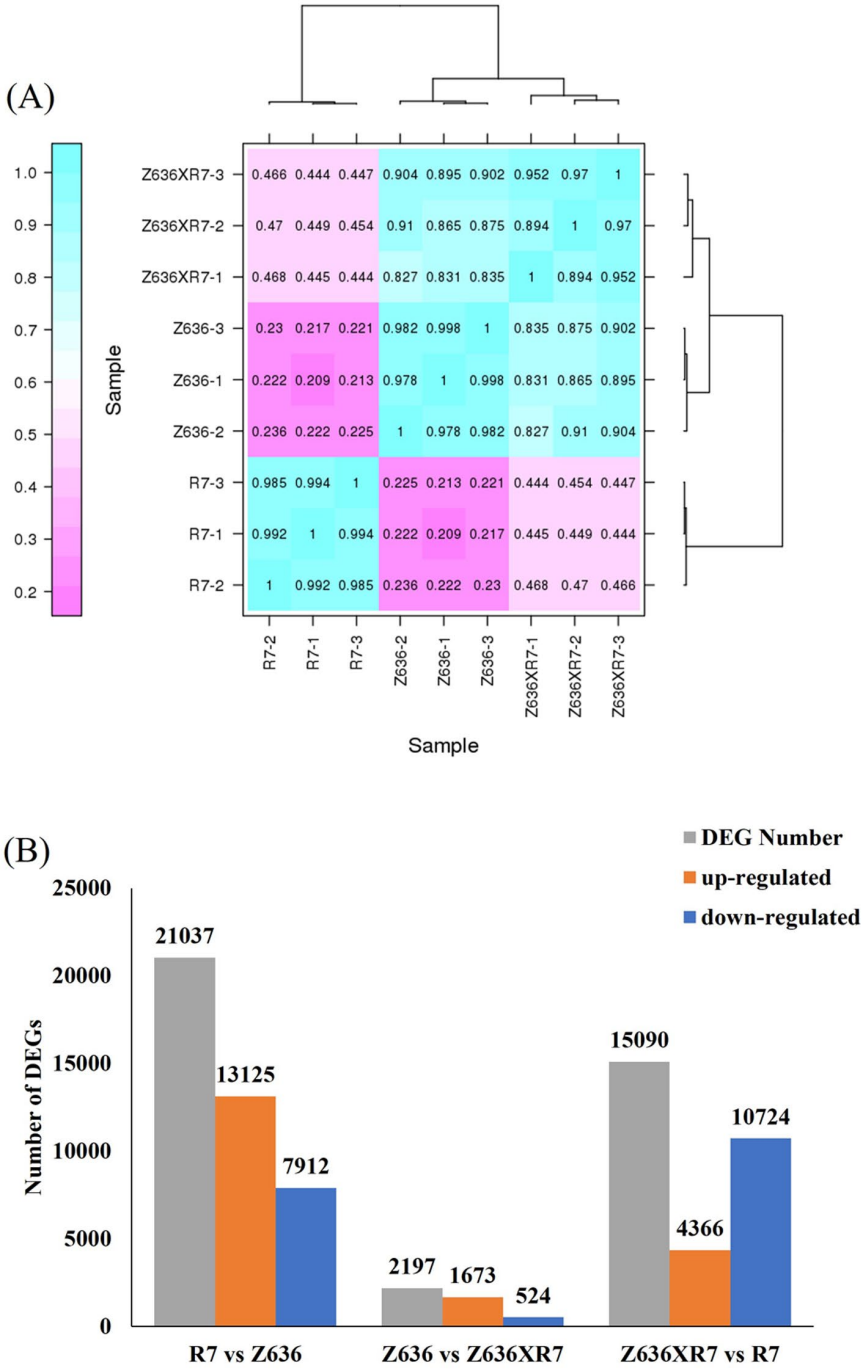
RNA-Seq analysis in R7, Z636, and Z636×R7. **A**, Hierarchical clustering heatmap of Pearson’s correlation coefficients between samples. **B**, Quantitative statistics of DEGs

To compare the transcriptional differences in developing grains among genotypes R7, Z636, and Z636×R7, three biological replicates were set up for each genotype, with a total of nine cDNA libraries being sequenced. After raw-read filtering and quality control, Q20 reached more than 95.29%, and Q30 (an error probability for base calling of 1% or 0.1%) reached more than 90.5% (Supplementary Table 2). The hierarchical clustering heatmap of Pearson’s correlation coefficients showed the highly correlated expression profiles among the biological replicates for each genotype (Fig. 3A). The correlation within the three genotype groups reached more than 0.89. The high-quality sequencing results and the strong positive correlation between replicate samples showed that these transcriptomic data could be used for subsequent analyses.

### Statistical and functional analyses of differentially expressed genes (DEGs)

In order to obtain reliable gene expression profiles, reads with log2 Fold Change ≥ 2, and Fold Change≥-2, and false discovery rate (FDR) < 0.01 were selected to identify the DEGs. As shown in Fig. 3B, total (up-/down-regulated) DEGs in the three comparison groups of R7 vs Z636, Z636 vs Z636×R7, and Z636×R7 vs R7 were 21,037 (13,125/7,912), 2,197 (1,673/524), and 15,090 (4,366/10,724), respectively.

DEGs were functionally annotated based on Gene Ontology (GO) term enrichment analysis. The GO terms, namely biological process (BP), cellular component (CC), and molecular function (MF), are shown in Supplementary Fig. 2. R7 vs Z636, Z636 vs Z636×R7, and Z636×R7 vs R7 comparisons were annotated and analyzed for GO function with respect to 16,651, 1,731, and 11,614 DEGs, respectively, and a total of 12,347, 1,250, and 8,547 DEGs were annotated, respectively. The DEGs in the three comparison groups were mainly concentrated in the term BP, in processes such as “metabolic process”, “cellular process”, “single organism process”, and “biological process regulation”, suggesting that genes involved in regulating physiological processes and cell metabolism play an important role in developing grains. Most DEGs in the term CC were enriched for “cell”, “cell part”, “membrane”, “membrane part”, and “organelles”, suggesting that these DEGs may be enriched in wheat endosperm cells and the intercellular cytoplasmic skeleton. With respect to the term MF, DEGs were mainly enriched for “binding”, “catalytically active”, and “transporter activity” processes, which affect the growth and development of wheat, enzymatic catalytic reactions, and ligand/receptor interactions.

In addition, Kyoto Encyclopedia of Genes and Genomes (KEGG) pathway enrichment was analyzed for all DEGs in each inter-genotype comparison, selecting the top 20 pathways with significant enrichment to display (Supplementary Fig. 3). A total of 16,651 DEGs in the R7 vs Z636 comparison were annotated to 10,406 KEGG pathways, which were mainly enriched with respect to “carbon metabolism”, “biosynthesis of amino acids”, “ribosomes”, “photosynthesis-antenna protein”, and “RNA transport”. The Z636 vs Z636×R7 comparison contained 1,731 DEGs annotated to 1,059 KEGG pathways, mainly enriched with respect to “starch and sucrose metabolism”, “amino sugar and nucleotide sugar metabolism”, “galactose metabolism”, and “pentose and glucuronate interconversion”. In the Z636×R7 vs R7 comparison, there were 11,614 DEGs annotated to 7,184 KEGG pathways, which were mainly enriched with respect to “photosynthesis-antenna protein”, “ribosome biogenesis in eukaryotes”, “carbon metabolism”, and “ribosome”, indicating that genes related to starch and sucrose metabolism, protein synthesis and processing, and hormone regulation had important regulatory roles in developing wheat grains.

### Differential expression of genes related to grain reserve polymers

**Fig. 4:**
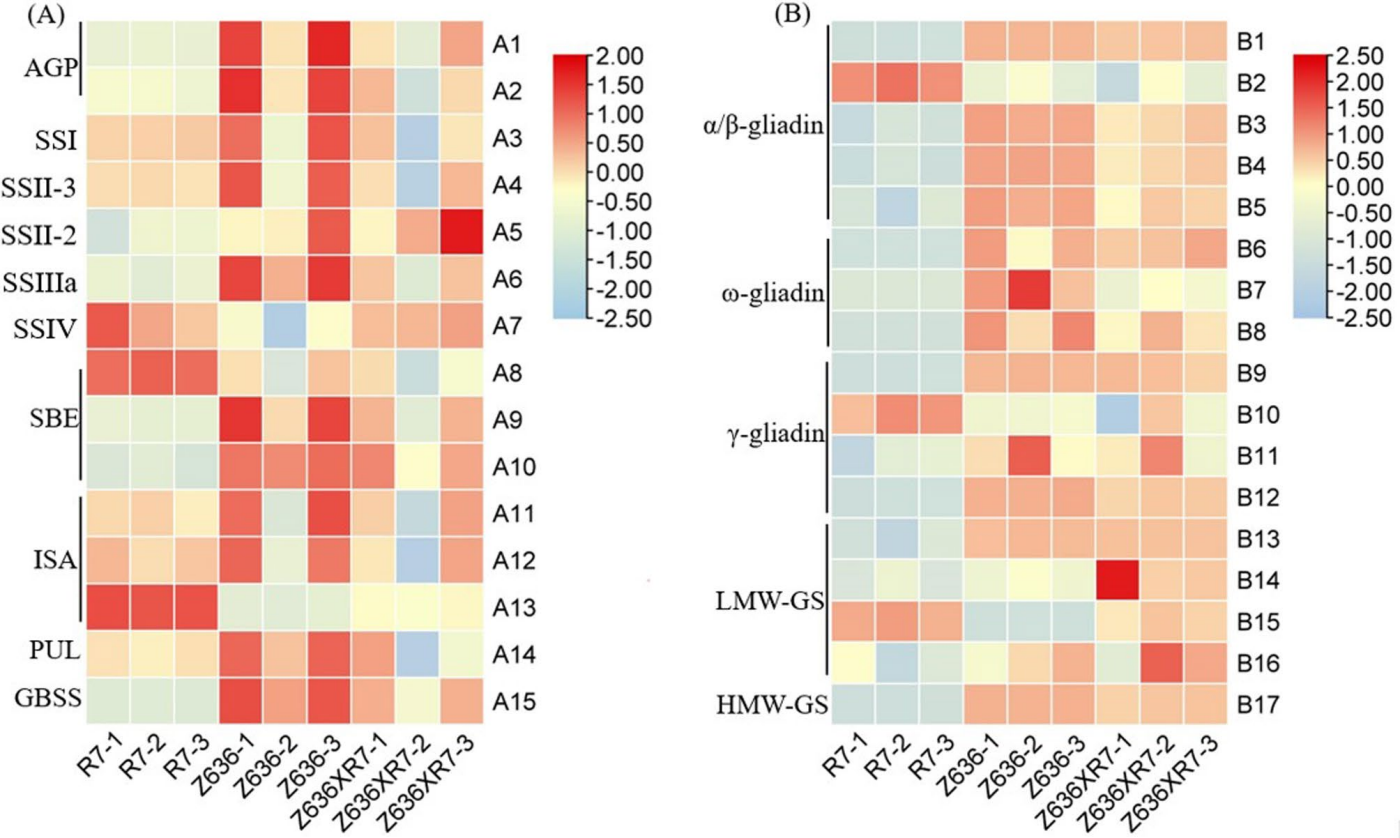
Expression patterns of starch synthase gene and storage protein gene. **A**, starch synthase genes. **B**, storage protein genes

The expression patterns of DEGs related to grain reserve polymers were investigated in developing grains of genotypes R7, Z636, and Z636×R7. Starch synthase genes, including *AGPase*, *SS*, *SBE*, and *DBE*, were identified in all differential cDNA libraries. The results indicated that the expression levels of starch synthase genes *AGP-L*, *AGP-S*, *SSII-2*, *SSIIIa*, *SBEII*, *SBEIII*, and *GBSSI* in Z636 were significantly higher than those in R7, but significantly lower than those in Z636×R7. Genes *SSI*, *SSII-3*, *ISAI*, *ISAII*, and *PUL* exhibited significantly higher expression in Z636 than in R7 or Z636×R7, whereas the expression levels of *ISAIII*, *SBEI*, and *SSIVa* genes in R7 were significantly higher than in Z636 or Z636×R7 (Fig. 4A, Supplementary Table 3).

When screening for genes encoding seed storage proteins, we found that gliadin and glutenin genes were differentially expressed between R7, Z636, and Z636×R7 (Fig. 4B, Supplementary Table 3). Seventeen glutenin- or gliadin-encoding DEGs were characterized in all libraries. The expression levels of genes encoding α/β-gliadin (TRITD0Uv1G108590), γ-gliadin (TRITD1Bv1G001870), and LMW-GS (TRITD1Bv1G008290) in R7 were significantly higher than those in Z636 or Z636×R7, whereas other seed protein-encoding DEGs showed significantly lower expression in Z636 or Z636×R7. The expression level of the gene encoding LMW-GS (TRITD1Av1G002790) in Z636×R7 was significantly higher than that in R7 or Z636. The α/β-, ω- and γ -gliadin results indicated that the expression patterns in Z636×R7 were similar to those in Z636, whereas their expression levels were significantly higher than those in R7 (Fig. 3B, Supplementary Table 3). The HMW-GS profile of Z636×R7 is composed of (1S^sh^x2.9 + 1S^sh^y2.3) and (1Bx6 + 1By8), which makes its glutenin expression level higher than that of Z636 and R7.

**Fig. 5:**
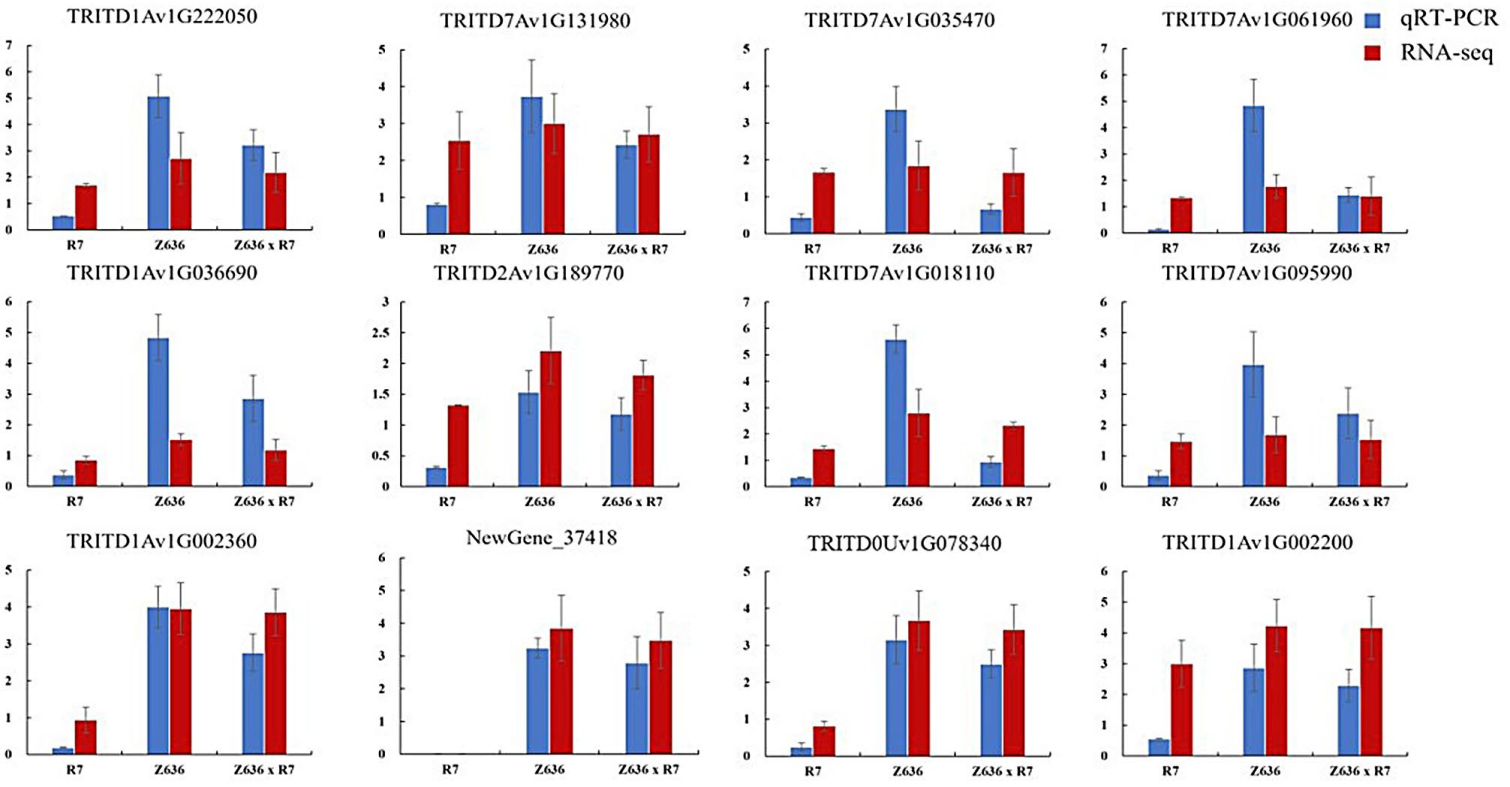
RT-qPCR validation of DEGs identified by RNA-Seq. The blue and red columns represent RT-qPCR and RNA-Seq data, respectively

In order to verify the reliability of RNA-Seq data, the expression patterns of 12 genes including eight starch synthase genes (*AGP-L*, *AGP-S*, *SSI*, *SSII-3*, *SSIIIa*, *SBEII*, *GBSSI*, and *ISAI)*, and four seed storage genes (*LMW-GS*, *HMW-GS*, *α-gliadin*, and *γ-gliadin*), were analyzed by quantitative reverse transcription-PCR (qRT-PCR). The IDs and primers of the selected genes are listed in Supplementary Tables 3 and Supplementary Tables 4 and the results of qRT-PCR analysis are shown in Fig. 5. The results of qRT-PCR showed that the expression levels of these genes in Z636×R7 were significantly lower than those in Z636 and higher than those in R7, which was consistent with the expression patterns derived from RNA-Seq data. Therefore, the RNA-Seq data were considered to be reliable for expression pattern analysis.

### Metabolome level and composition analysis

**Fig. 6:**
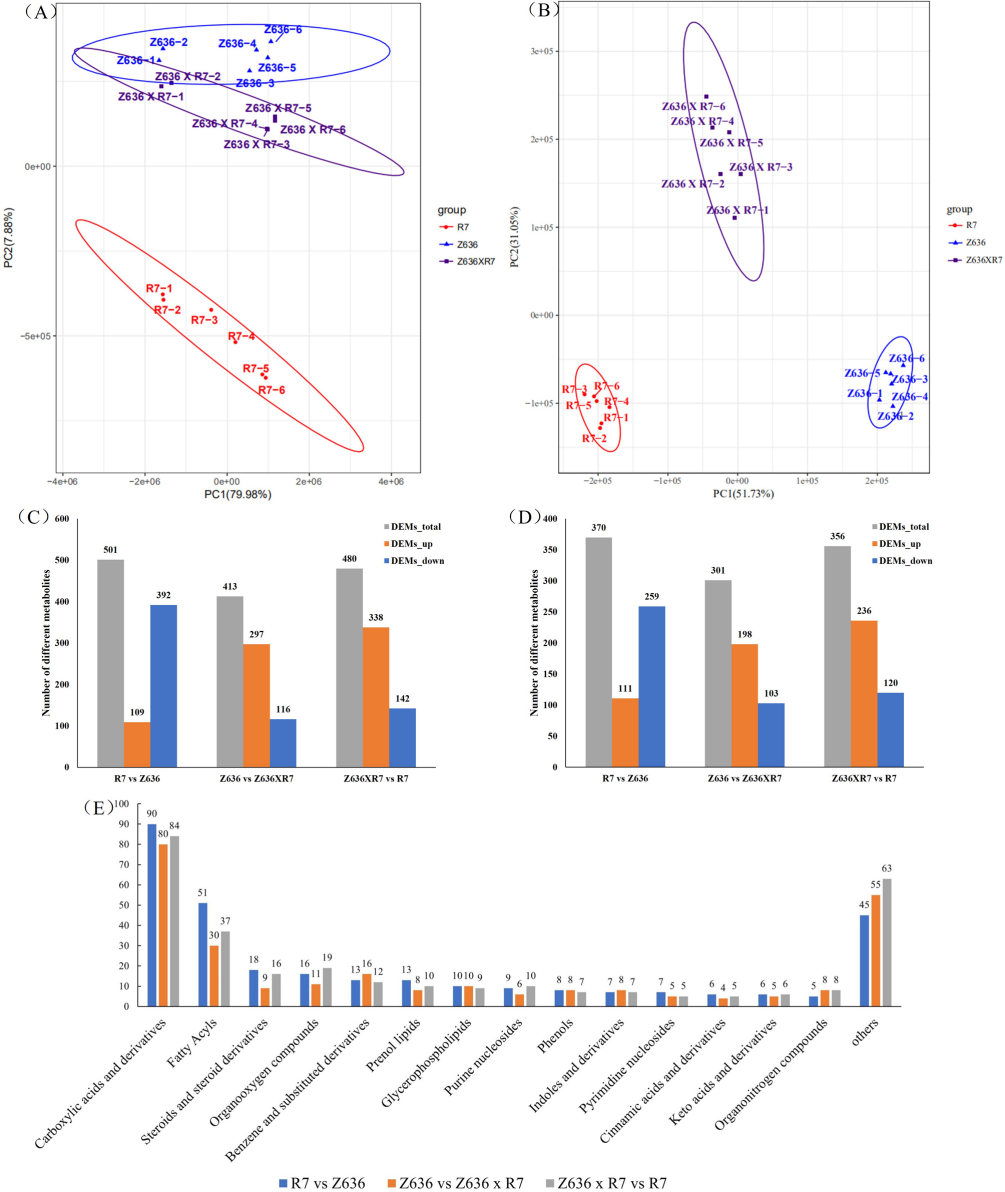
Metabolic variation in R7, Z636, and Z636×R7. **A**, **B**, Principal component analysis in positive ion mode and negative ion mode: the X-axis represents the first principle component (PC1), and the Y-axis represents the second principal component (PC2). **C**, **D**, Statistical analysis of the number of different metabolites in positive and negative ion modes. Gray is DEMs-total, blue is DEMs-upregulated, yellow is DEMs-downregulated. **E**, Category of differential metabolites

To construct a systematic profile of metabolic differences in R7, Z636, and Z636×R7, an untargeted metabolomic analysis was performed between R7 vs Z636, Z636 vs Z636×R7, and Z636×R7 vs R7. Principal component analysis (PCA) showed that the contribution rates of PC1 were 79.98% and 51.73%, and the contribution rates of PC2 were 7.88% and 31.05% in the positive and negative ion modes, respectively, of R7, Z636, and Z636×R7 (Fig. 6A and B). These results indicated that the differences between biological replicates during the experiment were small, and that there were differential metabolites between groups.

Differentially expressed metabolites (DEMs) were screened with a screening standard of VIP ≥ 1 and *P* value < 0.05. In the comparison R7 vs Z636, 501 and 370 DEMs were detected under positive and negative ion modes, respectively, of which 109 and 111 DEMs were upregulated, and 392 and 259 DEMs were downregulated, respectively. In the Z636 vs Z636×R7 comparison, 413 and 301 DEMs were detected under positive and negative ion modes, respectively, of which 297 and 198 were upregulated and 116 and 103 were downregulated, respectively. In the Z636×R7 vs R7 comparison, 480 and 356 DEMs were detected under positive and negative ion modes, respectively, of which 338 and 236 DEMs were upregulated and 142 and 120 DEMs were downregulated, respectively (Fig. 6C and D).

All DEMs identified in positive or negative ion modes are shown in the Fig. 6E and Supplementary Table 5. When comparing R7 vs Z636, a total of 871 DEMs were detected, of which 319 were known metabolites, which, in turn, belonged to 50 classes. A total of 714 DEMs were detected in the Z636 vs Z636×R7 comparison, of which 263 were known metabolites, which were divided into 45 classes. A total of 836 DEMs were identified in the Z636×R7 vs R7 comparison, of which 293 were known metabolites, belonging to 50 classes. Of these known metabolites, most were carboxylic acids and their derivatives, fatty acyls, steroids, and steroid derivatives, organic oxygen compounds, benzene and its derivatives, prenol lipids, glycerophospholipids, and purine nucleosides. Among these, carboxylic acids and their derivatives accounted for the highest numbers of metabolites, with t 90 (R7 vs Z636), 80 (Z636 vs Z636×R7), and 84 (Z636×R7 vs R7), respectively, followed by fatty acyl groups, with 51 (R7 vs Z636), 30 (Z636 vs Z636×R7), and 37 (Z636×R7 vs R7), respectively.

### KEGG enrichment analysis of DEMs

**Fig. 7:**
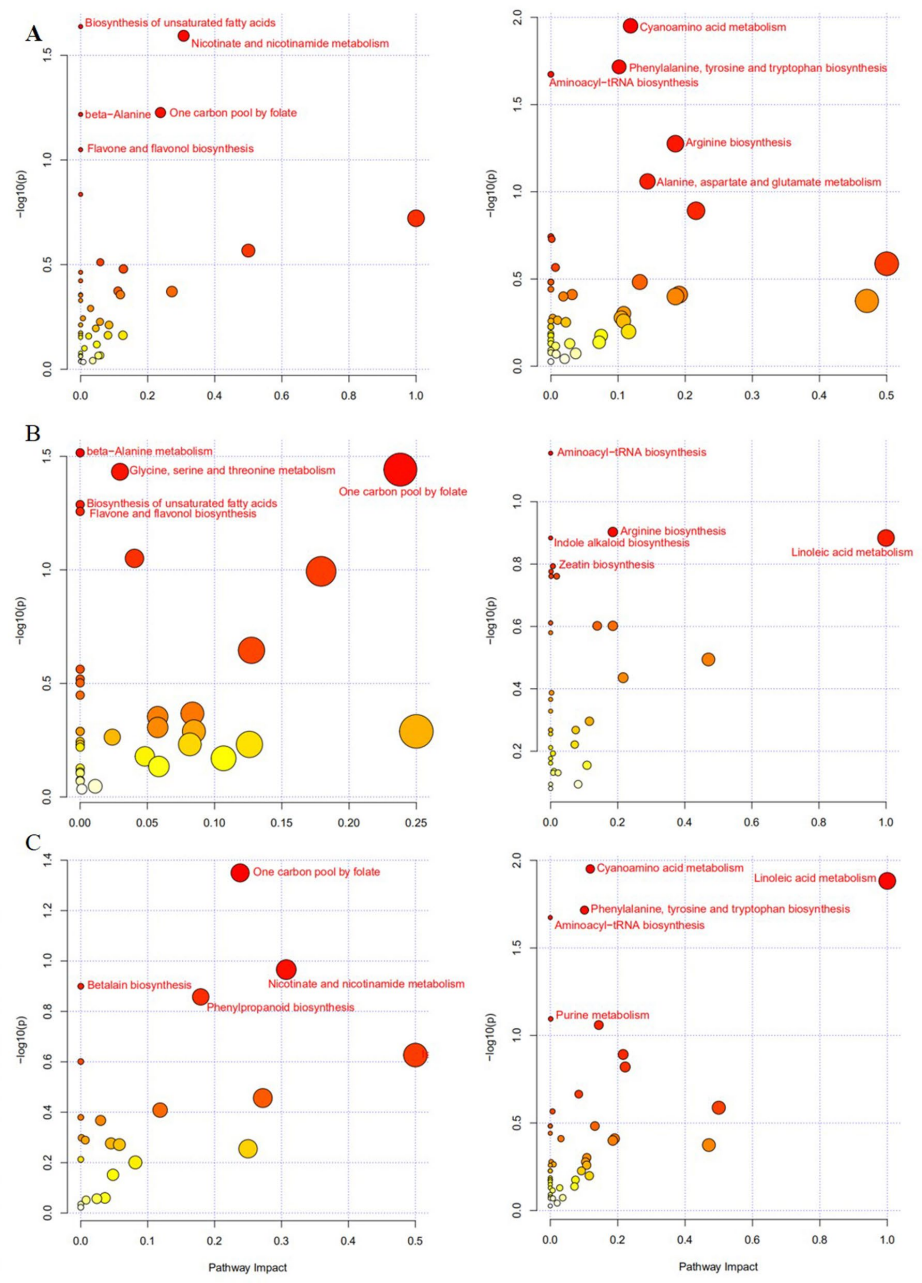
Pathway enrichment analysis of metabolites in positive ion modes and negative ion mode. **A**, R7 vs Z636. **B**, Z636 vs Z636×R7. **C**, Z636 vs Z636×R7. The left is positive ion modes and the right is negative ion mode

KEGG enrichment analysis of DEMs was performed to identify significantly enriched metabolic pathways. In positive and negative ion modes, the differential metabolites between R7 and Z636 were mainly enriched with respect to the biosynthesis of unsaturated fatty acids, niacin and nicotinamide metabolism, one-carbon pool by folate, β-alanine, flavonoid, and flavonol biosynthesis, carbamate metabolism, phenylalanine, tyrosine, and tryptophan biosynthesis, aminoacyl-tRNA biosynthesis, alanine, aspartic acid, and glutamate metabolism, etc. (Fig. 7A). The differential metabolites between Z636 and Z636×R7 in positive and negative ion mode were mainly enriched with respect to alanine metabolism, one-carbon pool by folate, glycine, serine and threonine metabolism, biosynthesis of unsaturated fatty acids, flavone and flavonol biosynthesis, aminoacyl-tRNA biosynthesis, arginine biosynthesis, etc. (Fig. 7B). The differential metabolites between Z636×R7 and R7 are mainly enriched with respect to the one-carbon pool by folate, niacin and nicotinamide metabolism, betaine biosynthesis, alanine metabolism, linoleic acid metabolism, phenylalanine, tyrosine and tryptophan biosynthesis, aminoacyl-tRNA biosynthesis, etc. (Fig. 7C).

### Correlation analysis of DEGs and DEMs associated with starch and amino acid synthesis

**Fig. 8:**
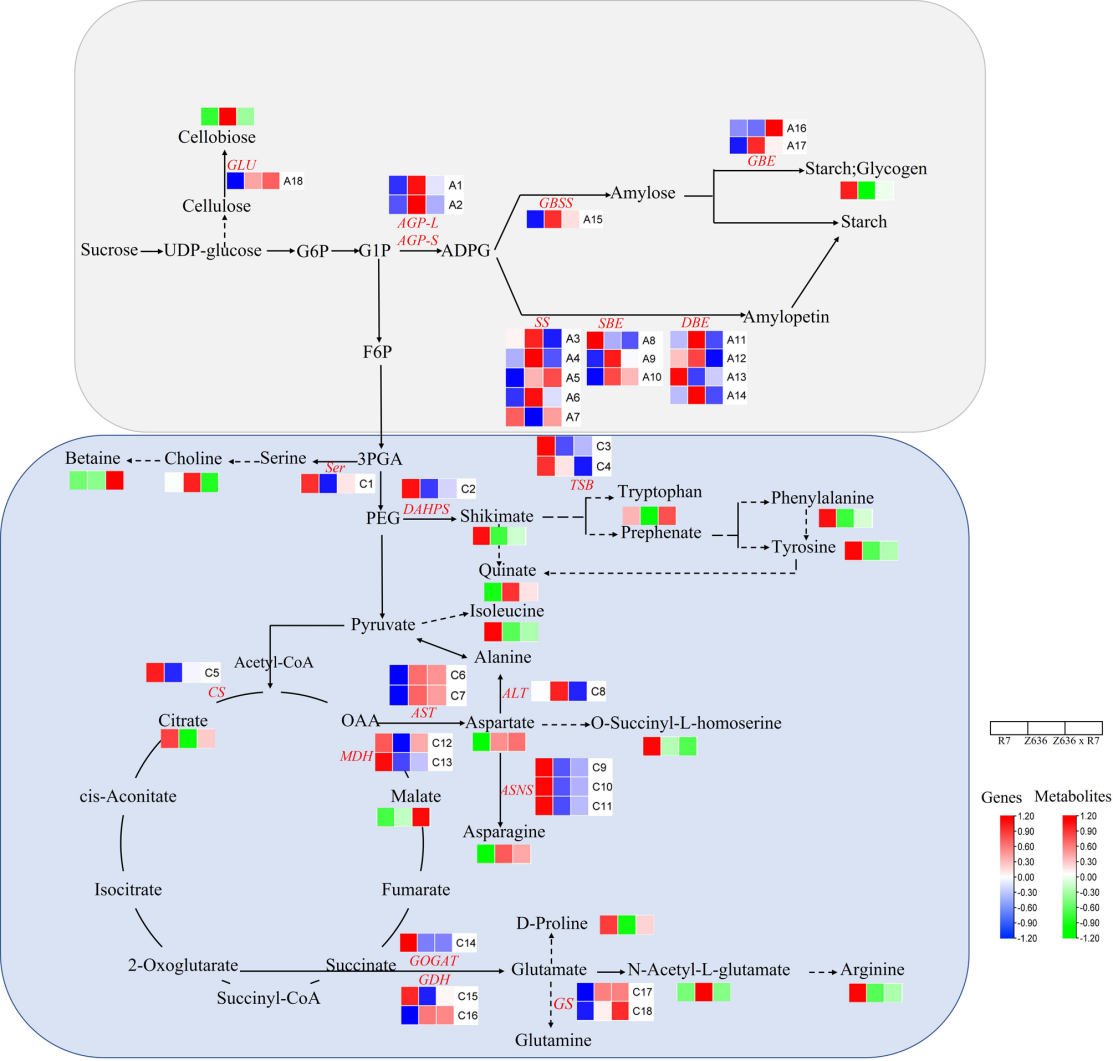
Metabolic pathways of R7, Z636, and Z636×R7. Black characters and red-green heatmaps represent the names and heatmaps of DEMs. Red characters and red-blue heatmaps represent the abbreviations and heatmaps of DEG annotation functions, and the three single cells represent R7, Z636, and Z636×R7 (metabolites and genes are in the same order). Solid arrows indicate that there are no intermediates between the two metabolites, and dotted arrows indicate that some intermediates are omitted from the pathway between the two metabolites

Based on GO and KEGG enrichment analyses, many DEMs and DEGs of R7, Z636 and Z636×R7 associated with starch and sucrose metabolism and with amino acid biosynthesis were identified (Fig. 8, Supplementary Table 3). The correlations between the content of cellobiose and the expression levels of genes *AGP-L*, *AGP-S*, *SS*, *SDE*, *DBE*, *GBSS*, α-1, 4-glucanase (*GBE*), and β-glucosidase in Z636×R7 were higher than those in R7 and lower than those in Z636×R7. The expression levels of genes associated with starch synthesis were positively correlated with the contents of amylose and total starch. GBE is a key enzyme in glycogen anabolism, catalyzing the formation of glycogen containing α-1,4-glycosidic and α-1,6-glycosidic bonds from ADP-glucose. In the current study, the correlation between *GBE* gene expression level and glycogen content in Z636×R7 was positive, but the correlations were negative in R7 and Z636.

With amino acid synthesis, the contents of amino acids such as homoserine, proline, arginine, and isoleucine in Z636 were significantly downregulated, relative to R7, to varying degrees, whereas the contents of aspartic acid and glutamic acid were significantly upregulated. Aspartate and glutamate are also important reserves for nitrogen supply. The contents of proline, arginine, tryptophan, and phenylalanine in Z636×R7 were significantly higher, but the contents of asparagine and glutamate were significantly downregulated. Compared with Z636×R7, the content of many differential metabolites in R7, such as isoleucine, homoserine, phenylalanine, arginine, lysine, etc., showed different degrees of increase, whereas the significantly down-regulated amino acid metabolites were mainly aspartic acid, asparagine, etc. These results suggest that the metabolic pathways of small molecular compounds containing carbon and nitrogen are changed after allopolyploidy, which may lead to changes in the content of macromolecular substances such as starch and protein.

In addition, DEGs associated with amino acid biosynthesis, such as phosphoserine transaminase (*SER*), tryptophan synthase (*TSB*), phospho-2-dehydrogen-3-deoxy heptanoate aldolase (*DAHPS*), alanine aminotransferase (*ALT*), asparagine synthetase (*ASNS*), glutamate synthase (*GOGAT*), and glutamate dehydrogenase (GDH1), were highly expressed in R7. It was also found that, for two DEGs responsible for encoding glutamate synthase (GOGAT), namely *GSr1* and *GSe2*, genotypes Z636 and Z636×R7 had significantly higher expression levels of *GSr1* than R7, whereas Z636×R7 had significantly higher gene expression levels of *GSe2* than either of its parents.

## Discussion

Wheat grain dry biomass contains 65–80% carbohydrates, mainly composed of starch [23, 24]. Biosynthesis of starch is accomplished by two types of enzymes, grain-binding starch synthase (GBSS) and starch synthase (SS) [25, 26]. Different expression levels of these starch synthase genes may affect starch synthesis and accumulation during grain filling [27]. Considerable progress has been made to date in the study of genes related to starch synthesis in tetraploid and hexaploid wheat [28], but little is known about the changes in the expression of genes involved in starch synthesis as a result of polyploidization.

The present omics and phenotypic study of Z636×R7, obtained from the cross between Z636 and R7, allowed analysis of the effect of polyploidization on the synthesis of seed storage polymers in wheat. Through the display of KEGG enrichment of DEGs, carbon metabolism, amino acid biosynthesis, RNA transport, starch and sucrose metabolism, amino and nucleotide sugar metabolism, galactose metabolism, pentose and glucuronic acid interconversion were the main metabolic pathways associated with differential gene enrichment. Some studies have shown that starch content and the expression level of starch synthase genes in synthetic hexaploid wheat are higher than in their parents, with synthetic hexaploid wheat inheriting the starch granule development and starch synthase gene expression pattern of the tetraploid parents [29]. In the current study, the expression levels of the starch biosynthesis genes *SSII*, *SSIIIa*, *SBEII*, *SBEIII*, and *GBSSI* in Z636×R7 were significantly higher than those in R7, but significantly lower than those in Z636. The patterns of starch synthase gene expression and starch content in Z636×R7 were more similar to Z636 than to R7, supporting the hypothesis that amphidiploid starch synthase gene expression is determined more by the tetraploid than by the diploid parent. Many studies have shown that allopolyploidy induces targeted sequence elimination, random structural changes, epigenetic and DNA methylation changes, and gene silencing of gene expression [30, 31]. The ADPase activity involved in starch biosynthesis in Z636×R7 may be restricted by the additional gene. The expression level of the corresponding starch biosynthesis gene in Z636×R7 was downregulated compared with Z636, which, in turn, affects the synthesis of amylose and amylopectin, which ultimately causes the starch content of Z636×R7 to be significantly lower than that of Z636.

Synthesis of seed storage proteins requires an adequate supply of amino acids, most of which are transported through the phloem, with the supply capacity of amino acids being strongly positively correlated with storage protein content [32]. Storage proteins are composed of storage protein particles known as protein body I and protein body II. Among these storage proteins, glutenins are mainly stored in protein body II, are rich in amino acids such as lysine, arginine, and glycine, and are readily decomposed by protease activity, whereas gliadins are mainly stored in protein body I, contain less lysine and are not readily degraded by trypsin [33]. Compared with the parent lines R7 and Z636, the content of seed storage proteins in Z636×R7 changed to different degrees, with the accumulation of the differential amino acid arginine decreasing in the amphiploid. Glutamine synthase (GS) is a key enzyme in plant nitrogen metabolism, responsible for the first step of ammonium assimilation and its conversion to glutamine [34, 35]. Glutamate can be converted into other amino acids by various transaminases and transported to wheat grains. In addition, the *GS* gene can be regulated to enhance the nitrogen reuptake capacity of grains. Some quantitative trait locus (QTL) analysis studies have found that *GS1* can regulate grain size [36] and that *GS2* expression is related to grain protein content [35]. In the current study, the *GSe2* expression level in Z636×R7 was higher than that of either of the two parents, a change that is conducive to increased protein synthesis and thus increased grain protein content in the amphidiploid.

Recent studies have shown that polyploidy can increase glutenin content by up-regulating glutenin biosynthesis, transport, and storage, and thus affecting the content of related essential amino acids [37]. Compared with R7, the expression levels of genes encoding HMW-GSs, LMW-GSs, α/β-, γ-, and ω-gliadins in Z636×R7 were significantly upregulated. RP-HPLC results showed that the total glutenin content of Z636×R7 was significantly higher than that of either of the two parents, whereas the total gliadin content was significantly higher than that of R7, but not significantly different from Z636. We speculate that the higher glutenin content of Z636×R7 than either parent might be due to the amphidiploid consisting of four HWM-GSs (1S^sh^x2.9 + 1S^sh^y2.3, 1Bx + 1By), so that the glutenin content of the polyploid is increased. Following hybridization of Z636 with R7 and subsequent spontaneous polyploidization, the alien *Aegilops* HMW-GSs content could be easily introduced into common wheat and improve the processing quality. The research results provided a basis for a greater in-depth understanding of the mechanism of wheat allopolyploid formation and its stable preservation, and also promoted the effective exploitation of high-value alleles from related species in wheat genetic improvement. Meanwhile, It provided new materials for the transfer of good genes into cultivated wheat.

Due to the limited capacity to correctly identify the transcript of *Ae. sharonensis* and the only partial identification of plant metabolites, the interpretation of the results may be biased.

## Conclusion

The allopolyploidization has led to the down-regulated expression of genes related to starch synthesis in amphidiploid, resulting in delayed starch accumulation and a prolonged seed development process. However, the expression of the *GSe2* gene in amphidiploid was higher than that of the two parents, which was beneficial to protein synthesis and thus increased protein content and eventually led to the changes in the synthesis of seed reserve polymers. The current study revealed that allopolyploids have profound effects on the synthesis of seed reserve polymers.

## Materials and methods

### Plant materials

Seed of *Aegilops sharonensis* (R7, S^sh^S^sh^, 2n = 14) PI584388 were kindly provided by the United States Department of Agriculture-Agricultural Research Service (USDA-ARS) (https://www.ars-grin.gov/), *Triticum turgidum* ssp. *durum* (Z636, AABB, 2n = 28) was provided by the Triticeae Research Institute, Sichuan Agricultural University, China, and the amphidiploid (AABBS^sh^S^sh^, 2n = 42) of *Triticum turgidum* subsp. *Durum* × *Ae. sharonensis* was generated in our previous work [22]. All the plant materials were grown in greenhouses in 2019 and fields in 2021 and are preserved in the Triticeae Research Institute of Sichuan Agricultural University.

### Sodium dodecyl sulfate-polyacrylamide gel electrophoresis (SDS-PAGE)

Storage proteins were extracted from the non-germ portion of individual mature seeds as described previously [38]. The extracts were heated at 95℃ for 5 min and centrifuged at 10,621×g for 10 min. The supernatant was loaded onto a 10% (w/v) SDS-PAGE gel and electrophoresis was carried out [6, 39].

### Multi-color fluorescent in-situ hybridization (mc-FISH)

The methods described by [40] were used to prepare mitotic metaphase chromosome spreads. The probes Oligo-pSc119.2-1, Oligo-pTa535-1, and (AAG)_6_ (Sangon, Shanghai, China) (Supplementary Table 1) were used in mc-FISH experiments to characterize alien chromosomes of *Ae. sharonensis.* The probes of Oligo-pSc119.2-1 and Oligo-pTa535-1 were developed based on repeat sequences available in NCBI (https://www.ncbi.nlm.nih.gov/*)*, and can provide a signal to distinguish the *Ae. sharonensis* chromosomes from those of wheat and other wild species [41]. Oligo-pSc119.2-1, Oligo-pTa535-1, and (AAG)_6_ were 5’ end-labeled with 6-carboxyfluorescein (6-FAM), 6-carboxy tetramethylrhodamine (TAMRA), or Cy5, respectively. The mc-FISH analysis was performed according to the procedure described by [42]. Images were obtained using an epifluorescence microscope (BX51, Olympus, Tokyo, Japan).

### Reverse-phase high-performance liquid chromatography (RP-HPLC) analysis of gluten proteins

RP-HPLC was used to separate and compare the contents of glutenins (HMW-GS, LMW-GS) and gliadins (ω, α/β, γ) of R7, Z636, and Z636×R7. The extraction method for glutenins and gliadins was carried out as previously described [43, 44]. The gluten proteins from each genotype were extracted from three independent flour samples. The glutenins (HMW-GS and LMW-GS) were extracted from 45 mg of flour from each genotype at room temperature by adding 1 mL of a mixture of 0.3 mol/L NaI and 7.5% 1-propanol and carrying out gentle shaking for 10 min. The mixture was centrifuged at 17,949×g for 10 min to recover the pellet. The pellet was suspended in 1 mL of 70% ethanol with gentle agitation at room temperature for 30 min. The suspension was then centrifuged at 17,949×g for 10 min to collect the pellet for suspension in 1 mL of 55% isopropanol. The suspension was then incubated at 65℃ for 30 min with periodic agitation every 5 min, followed by centrifugation at 17,949×g for 10 min. This step was repeated once to recover the pellet, with the pellet being suspended in 500 µL of 50% isopropanol, 80 mmol/L Tris-HCl (pH 8.0), and 1% (w/v) dithiothreitol and the suspension incubated at 65℃ for 1 h, after which 1% (w/v) 4-vinylpyridine was added to the mixture, which was incubated at 65℃ for 30 min. Subsequently, the suspension was centrifuged at 17,949×g for 10 min to collect the supernatant, which represented the glutenin fraction. The gliadins were extracted from 45 mg flour in 1 mL of 70% ethanol, with periodic agitation, for 1 h. The suspension was then centrifuged at 17,949×g for 10 min, and the supernatant was retained as the gliadin fraction.

The gluten protein fractions were then filtered through a 0.45-µm pore size nylon filter before separating on an RP-HPLC, after loading 20 µL of supernatant onto the C18 column (Cat. 880,995–902, ZORBAX300SB-C18; 4.6 mm×250 mm, 5 μm particle size; Agilent Technologies, Palo Alto, CA, USA). The RP-HPLC was conducted with an Agilent 1260 Infinity LC instrument (Agilent Technologies, Palo Alto, CA, USA). The separation conditions for gliadins began with 79% of mobile phase A (0.06% trifluoroacetic acid in ultra-pure water) and 21% of mobile phase B (0.06% trifluoroacetic acid in acetonitrile) at 0 min, followed by 52% A and 48% B at 55 min, and 79% A and 21% B at 60 min to achieve the separation of all gliadins. The isolation of glutenins began with 75% A and 25% B at 0 min, followed by 52% A and 48% B at 45 min, and 75% A and 25% B at 50 min until all the glutenins were separated. The separation conditions were set to 60℃ for column temperature and 1 mL/min for flow rate, using a diode array detector (DAD) at a wavelength of 210 nm. There was a 10-min pause between runs to allow the column to be flushed with methyl alcohol. The contents of individual glutenins and gliadins were calculated by combining the related peak areas in the chromatograms.

### Starch analysis

The total starch, amylopectin, and amylose contents of R7, Z636 and Z636×R7 were measured using the Total Starch Assay Kit and Amylose/Amylopectin Assay Kit (Megazyme, Bray, Ireland), according to the manufacturer’s protocols. The final contents of total starch, amylopectin, or amylose in each genotype were determined using the data from three independent flour sample replicates.

### Total RNA isolation and transcriptome sequencing

The developing grains of R7, Z636, and Z636×R7 were harvested at 15 days post-anthesis (DPA), transferred immediately into liquid nitrogen, and stored at -80℃ until RNA was to be extracted. Total RNA was isolated using the Plant RNA Kit (Biofit, Chengdu, China), in accordance with the manufacturer’s protocol. The RNA concentration was measured using the NanoDrop 2000 spectrophotometer (Thermo Scientific, Waltham, MA, USA). RNA integrity was assessed using the RNA Nano 6000 Assay Kit of the Agilent 2100 Bioanalyzer (Agilent Technologies, Palo Alto, CA, USA). For RNA-Seq (RNA-sequencing), transcriptomicanalysis and metabolomic analysis of the three biological replicates of each sample, analyses were sub-contracted to BioMarker Technologies (Beijing, China), with RNA-Seq being conducted on the Illumina HiSeq high-throughput sequencing platform (HiSeq 2000, Illumina, San Diego, CA, USA).

### Transcriptome assembly, annotation, and differential expression analysis of the genes

Raw data in the Fast Q format were first processed through Perl script developed in-house. In this step, clean data were obtained by removing from the raw data reads containing adapters, poly-N sequences, and low-quality sequence reads. Simultaneously, Q20, Q30, GC-content, and sequence duplication levels of the clean data were determined. All the downstream analyses were based on clean data of high quality. The clean reads were then mapped to the reference genome sequence of *Triticum_turgidum*_ssp._*durum* (https://www.interomics.eu/durum-wheat-genome); only reads with a perfect match or with one mismatch were further analyzed and annotated based on the reference genome, using the HISAT2 V2.1.0 tool *(daehwankimlab.github.io)*.

Gene function was annotated based on the following databases: Pfam (Protein family) (https://pfam.xfam.org/); KO [Kyoto Encyclopedia of Genes and Genomes (KEGG) Ortholog database (https://www.genome.jp/kegg/ko.html); GO (Gene Ontology) (http://www.geneontology.org); and Swiss-Prot (a manually annotated and reviewed protein sequence database) (https://www.uniprot.org/). Quantification of the gene expression levels was achieved by fragments per kilobase of transcript per million fragments mapped (FPKM).

The differential expression analysis between two genotype groups was completed using the R package ‘DEseq’. The resulting *P*-values were adjusted using the approach of Benjamini and Hochberg for controlling the false discovery rate (FDR). Genes with log2 Fold Change ≥ 2 and FDR < 0.01 were assigned as DEGs.

### Metabolite extraction, detection, and bioinformatic analysis

The samples of developing grain from each of the three genotypes were extracted for metabolome analysis. Briefly, 50 mg of each sample (with three replicates of each sample) were placed in an Eppendorf tube, to which was added 1 mL extraction solvent (methanol: acetonitrile: water = 2:2:1 [v/v/v]). Each sample was then homogenized in a ball mill for 4 min at 45 Hz, then ultrasonically extracted for 5 min, the container being cooled by incubation in ice water. After three rounds of homogenization, the sample was incubated for 1 h at − 20℃ to precipitate proteins. The sample was then centrifuged at 15,294 ×g for 15 min at 4℃, after which the supernatant (500 µL) was transferred into a fresh Eppendorf tube. The supernatant was evaporated to dryness in a vacuum concentrator without heating and 100 µL extraction solvent (acetonitrile: water = 1:1 [v/v]) were added to re-suspend the residue. The sample was then vortexed for 30 s, sonicated for 10 min (in a 4℃-water bath), and centrifuged for 15 min at 15,294 ×g at 4℃. Afterward, the supernatant (60 µL) was transferred into a fresh 2-mL liquid chromatography/mass spectrometry (LC/MS) glass vial. Lastly, 10 µL aliquots from each of the three independent replicates of each sample were then taken and pooled as quality control (QC) samples and 60 µL of supernatant were taken for an ultra-high-performance liquid tandem chromatography-quadrupole time-of-flight mass spectrometry (UHPLC-QTOF-MS) analysis.

The LC/MS system for metabolomic separation and detection was composed of ultra-high performance liquid chromatography (UPLC Acquity I-Class PLUS, Waters, USA) and high resolution mass spectrum (UPLC Xevo G2-XS QTOF, Waters, USA). The column used was Acquity UPLC HSS T3 (1.8 μm particle size, 2.1 × 100 mm) (Waters, USA). For the positive and negative ion modes, the mobile phases were A (0.1% formic acid in water) and B (0.1% formic acid in acetonitrile) and the injection volumes were 1.5 µL (positive) or 1 µL (negative). The TripleTOF mass spectrometer (Waters, USA) was used for its ability to acquire MS/MS spectra on an information-dependent basis during an LC/MS experiment. In this mode, the acquisition software (Analyst TF 1.7, AB Sciex) continuously evaluates the full-scan survey MS data as it collects and triggers the acquisition of MS/MS spectra, depending on preselected criteria. In each cycle, 12 precursor ions with intensities < 100 ppm were chosen for fragmentation at a collision energy (CE) of 30 V (15 MS/MS events with product ion accumulation time of 50 ms each). The electrospray ionization source conditions were set as follows: ion source gas 1 at 60 psi, ion source gas 2 at 60 psi, curtain gas at 35 psi, and Ion Spray Voltage Floating (ISVF) at 5,000 V value of OPLS-DA and the univariate statistical analysis of the t-test P-value to screen differentially expressed metabolites (DEMs) among different genotype groups for comparison. The classification and pathway information of the identified compounds are searched by using the datebases KEGG, Human Metabolome Database (HMBD), and LIPID MAPS. The screening criteria log2 Fold Change ≥ 1.2, P-value < 0.05, and VIP > 1 were taken into account to identify the DEMs between groups. Gene Ontology enrichment analysis of the DEMs was executed by the ‘goseq’ R package, based on the Wallenius non-central hyper-geometric distribution, which can adjust for gene length bias in DEGs [45]. The KEGG pathway enrichment analysis of the DEGs and DEMs was carried out using the KOBAS software [46]. The heatmap was created using the TBtool V0.66839 software [47].

### Real-time quantitative PCR (RT-qPCR) validation

The expression levels of 12 genes encoding components of HMW-GSs, gliadins, LMW-GSs, and starch were quantified by qRT-PCR to verify the RNA-Seq data. First-strand cDNA was synthesized using the PrimeScript™ RT Reagent Kit with gDNA Eraser (Takara, Dalian, China), and qPCR was carried out with SYBR® Premix Ex *Taq™* II (Takara, Beijng) on a CFX 96 Real-Time System (Bio-Rad, Hercules, CA, USA). The CFX Manager software was used to analyze the qPCR data and to calculate relative expression with the 2^−△△Ct^ method. The wheat actin and glyceraldehyde 3-phosphate dehydrogenase (GAPDH) genes were used as internal reference genes to normalize the relative expression of candidate genes.

### Statistical analysis

Analysis of variance (ANOVA) with One Way ANOVA was conducted. IBM SPSS statistical software (SPSS Inc., Chicago, IL, USA) and GraphPad Prism 8 were used for statistical analysis and mapping, and *P* < 0.05 was considered to be statistically significant.

### Electronic supplementary material

Below is the link to the electronic supplementary material.

Supplementary Material 1: **SFig. 1** The full gel of HMW-GS composition determined by SDS-PAGE

Supplementary Material 2: **SFig. 2** GO functional annotation of DEGs

Supplementary Material 3: **SFig. 3** Histogram of KEGG enrichment of differently expressed genes

Supplementary Material 4: **STable 1** Oligonucleotide probes for fluorescence in-situ hybridization (FISH) analysis

Supplementary Material 5: **STable 2** Statistical results of RNA sequencing data quality

Supplementary Material 6: **STable 3** List of gene IDs with differentially expressed genes related to starch, storage protein and amino acid synthesis

Supplementary Material 7: **STable 4** Primers used in RT-qPCR

Supplementary Material 8: **STable 5** Classification and quantity of DEMs identified in metabolomic analysis

## Data Availability

The wheat actin and glyceraldehyde. All data generated or analyzed during this study are available in Supplementary Materials.
