# Supplementary figures and images for: Multi-omic analysis reveals the effects of interspecific hybridization on the synthesis of seed reserve polymers in a Triticum turgidum ssp. durum × Aegilops sharonensis amphidiploid
Source: BMC Genomics. 2024 Jun 20;25:626. doi: 10.1186/s12864-024-10352-9 (PMC11188524; doi:10.1186/s12864-024-10352-9)

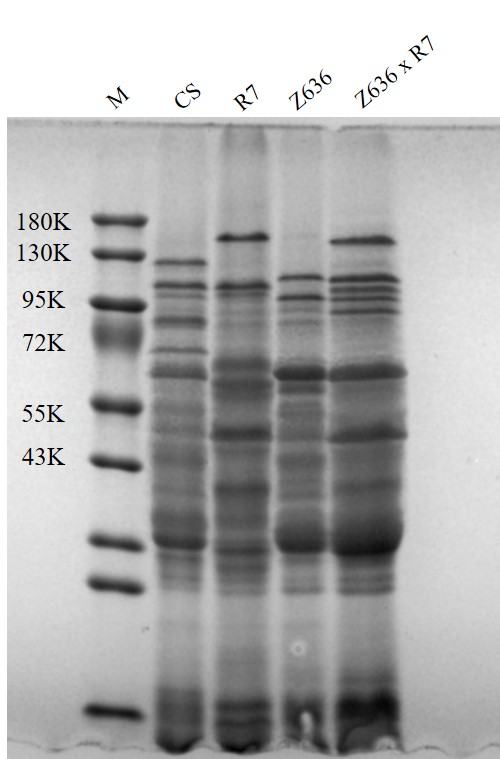


**Supplementary Figure 1**. The full gel of HMW-GS composition determined by SDS-PAGE

Supplement: Supplementary file 1 — Supplementary Material 1: SFig. 1 The full gel of HMW-GS composition determined by SDS-PAGE [file 12864_2024_10352_MOESM1_ESM.docx]

**
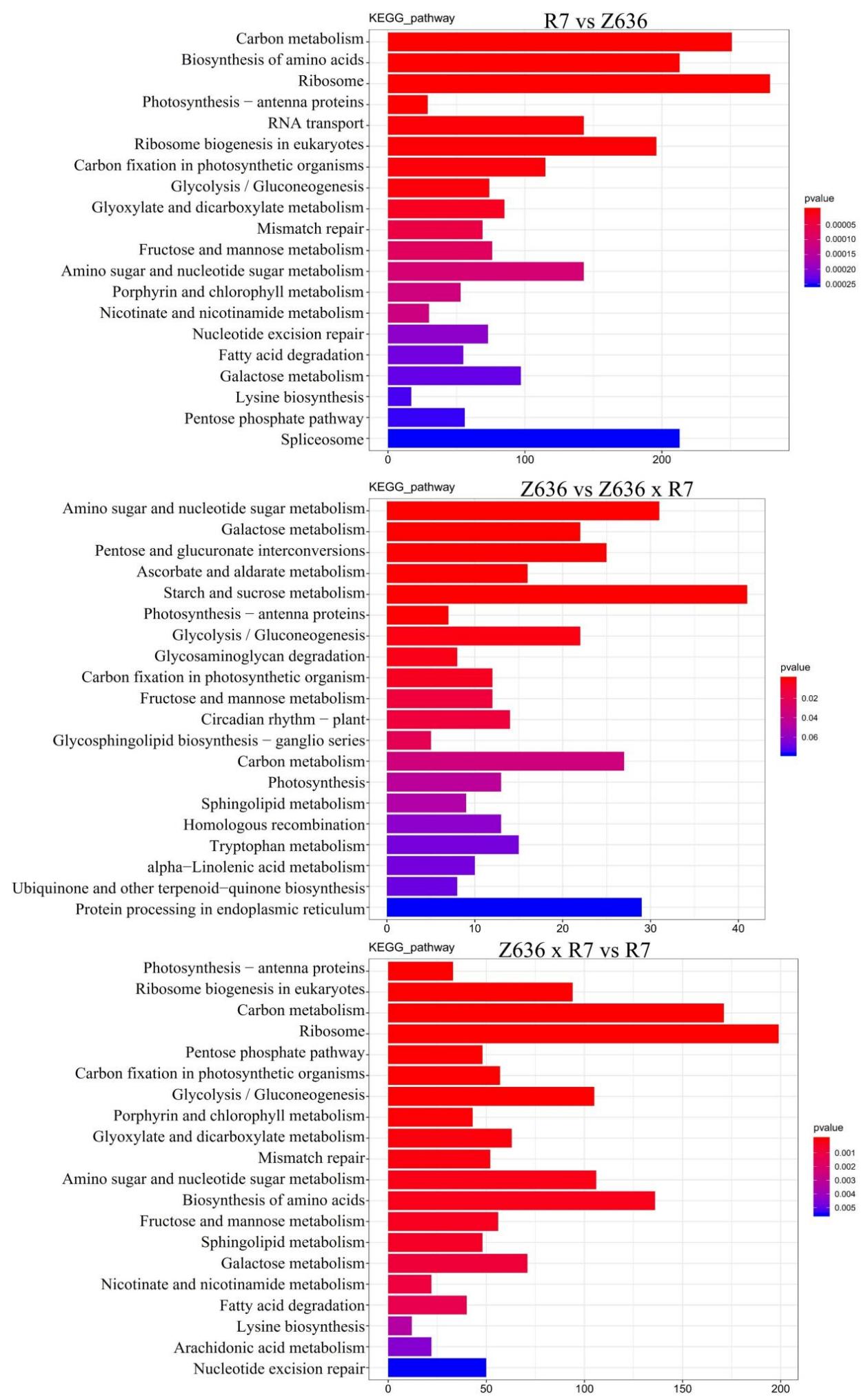
**

**Supplementary Figure 3.** Histogram of KEGG enrichment of differentlly expressed genes.

Supplement: Supplementary file 3 — Supplementary Material 3: SFig. 3 Histogram of KEGG enrichment of differently expressed genes [file 12864_2024_10352_MOESM3_ESM.docx]
