# Supplementary material for: Multi-omic analysis reveals the effects of interspecific hybridization on the synthesis of seed reserve polymers in a Triticum turgidum ssp. durum × Aegilops sharonensis amphidiploid
Source: BMC Genomics. 2024 Jun 20;25:626. doi: 10.1186/s12864-024-10352-9 (PMC11188524; doi:10.1186/s12864-024-10352-9)
Supplement: Supplementary file 2 — Supplementary Material 2: SFig. 2 GO functional annotation of DEGs [file 12864_2024_10352_MOESM2_ESM.docx]

**
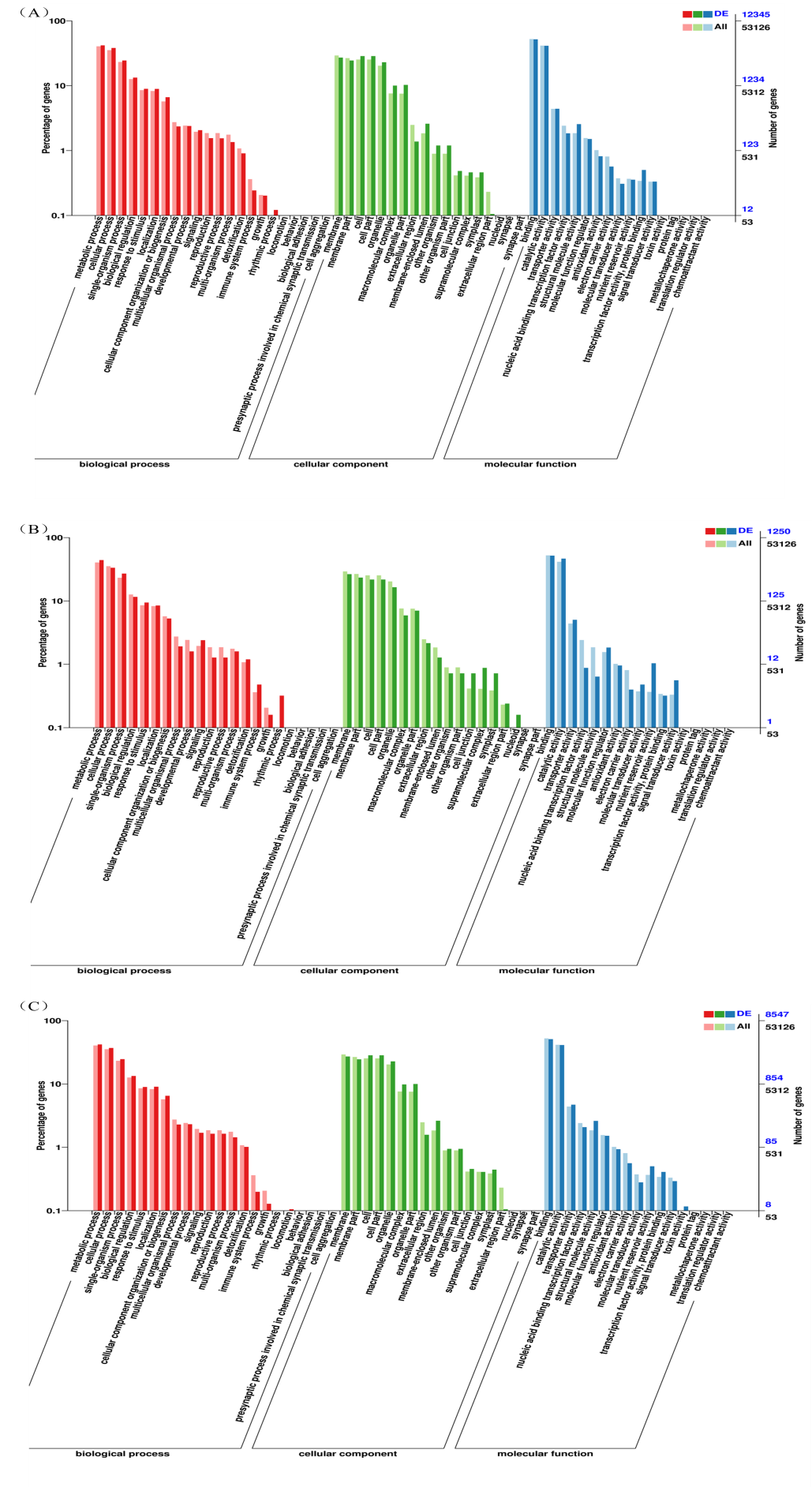
**

**Supplementary Figure 2.** GO functional annotation of DEGs. **A**, GO functional annotation of R7 vs Z636. **B**, GO functional annotation of Z636 vs Z636×R7. **C**, GO functional annotation of Z636×R7 vs R7.
